# Correlation of IL-6 secretion and hyponatremia with the use of CD19+ chimeric antigen receptor T-cells 

**DOI:** 10.5414/CN109872

**Published:** 2019-10-31

**Authors:** Brianne N. Dixon, Ryan J. Daley, Larry W. Buie, Meier Hsu, Jae H. Park, Renier J. Brentjens, Terence J. Purdon, Sheron Latcha

**Affiliations:** 1 Department of Pharmacy,; 2 Department of Epidemiology and Biostatistics,; 3 Department of Medicine, and; 4 Department of Immunology, Memorial Sloan Kettering Cancer Center, New York, NY, USA

**Keywords:** IL-6, hyponatremia, CD19+ CAR T-cells

## Abstract

Background: Various studies have demonstrated that interleukin-6 (IL-6) activates the central magnocellular arginine vasopressin (AVP)-secreting neurons in the brain to produce non-osmotic, non-volume-mediated increases in AVP. The most common toxicity of CD19+ chimeric antigen receptor (CAR) T-cells is cytokine release syndrome, which is related to increased levels of IL-6. This study will evaluate the correlation of IL-6 levels with hyponatremia in patients receiving CD19+ CAR T-cells. Materials and methods: This is a single-center retrospective analysis of adult patients who received CD19+ CAR T-cells for the treatment of relapsed/refractory acute lymphoblastic leukemia (ALL). Results: Hyponatremia, defined as a serum sodium (Na) ≤ 135 mEq/L, occurred in 31 (61%) patients. A change in Na > 7 mEq occurred in 32 (63%) patients, and the median lowest Na was 133 mEq/L (interquartile range (IQR): 131 – 136)). There was an inverse linear relationship between IL-6 levels and lowest Na (p = 0.001). Overall, per 10-fold increase in IL-6, Na decreased by an average of 2.68 mEq/L. Conclusion: Hyponatremia is common in patients who received CD19+ CAR T-cells. There is an inverse linear relationship between IL-6 levels and nadir Na (p = 0.001). Further studies will be needed to confirm a causative relationship between IL-6 levels and hyponatremia following CD19+ CAR T-cell infusion.

## Introduction 

### Background 

Animal and human studies have demonstrated that interleukin-6 (IL-6) activates the central magnocellular arginine vasopressin (AVP)-secreting neurons in the brain to produce non-osmotic, non-volume-mediated increases in AVP levels [1, 2, 3]. Hyponatremia in a patient with a normal volume status and a low serum osmolarity is referred to as the syndrome of inappropriate antidiuretic hormone (SIADH). IL-6-stimulated AVP secretion in euvolemic children has been shown to cause hypo-osmolar hyponatremia [4]. In ultramarathon runners who experienced exercise-associated hyponatremia (EAH), there was a significant positive correlation between IL-6 and AVP levels [5]. 

Chimeric antigen receptor (CAR) T-cells are an adoptive cellular therapy by which autologous or allogeneic donor T-cells are collected and then genetically reengineered to produce immune cells with direct anti-tumor activity. CD19+-targeted CAR T-cells have demonstrated high anti-tumor efficacy in patients with B-cell malignancies [6]. When the CAR T-cells are infused and encounter tumor cells, a cascade of cytokine-mediated events follows as a result of CAR T-cell activation, tumor lysis syndrome, and macrophage activation. Cytokine release syndrome (CRS) is the most common adverse effect following CAR T-cell infusion [7]. Upwards of 24 cytokines have been extensively studied in association with CRS [8]. As oncologists looked for a means to control CRS, IL-6 has garnered significant interest given the availability of tocilizumab. Tocilizumab is a humanized antibody to the IL-6 receptor that is Food and Drug Administration (FDA) approved for the treatment of CRS. It has proven to be effective in inhibiting some of the significant clinical toxicities associated with CRS [9]. 

### Aim 

The aims of this study are to report on the rate of hyponatremia in patients who have received CD 19+ CAR-T cell therapy and to examine the relationship between IL-6 levels and hyponatremia. 

## Materials and methods 

After obtaining approval from the Institutional Review Board at Memorial Sloan Kettering Cancer Center, we obtained retrospective data on all patients age ≥ 18 years who received CD19+ CAR T-cells with the CD28-ζ domain for treatment of relapsed/refractory acute lymphocytic leukemia (ALL) between July 1, 2013, and March 3, 2016. Data collection included serum sodium (Na), IL-6 levels, and tocilizumab treatment dates. Hyponatremia was defined as a serum Na < 135 mEq/L. Nadir Na value was the lowest measured Na after infusion of CAR T-cells. The IL-6 level was measured within a 24-hour period prior to the nadir Na value. Patients were excluded if they did not have a measured IL-6 level within 24 hours prior to nadir Na. 

### Statistical methods 

The distribution of serum Na on the day of nadir Na, the incidence of hyponatremia, defined as a serum Na < 135 mEq/L, demographic, clinical, and treatment characteristics were summarized descriptively for the cohort using frequencies, percentages, medians, and ranges. The change in serum Na is the difference between post-infusion nadir Na and pre-infusion serum Na measurement, summarized as a proportion of patients with a change in Na > 7 mEq/L. 

The relationship between serum Na level on the day of nadir Na and IL-6 level within 24 hours prior to day of nadir Na was shown as a scatter plot. A linear regression model of nadir serum Na as the dependent variable and IL-6 as the independent variable was fitted and overlaid on the scatter plot. A base 10 logarithmic transformation was applied to IL-6 such that the coefficient can be interpreted as the change in serum Na per 10-fold increase in IL-6 level. 

The difference in median IL-6 between hyponatremic and non-hyponatremic patients was also compared using the Wilcoxon rank sum test. The odds ratio was estimated from a logistic regression model of hyponatremia and continuous IL-6 and can be interpreted as the likelihood of hyponatremia per 10-fold increase in IL-6. 

## Results 

Patient demographics and disease characteristics are shown in Table 1. Of the 51 patients included in this analysis, 38 (75%) were male, and the median age was 44 years (range 23 – 74 years). All patients were treated for relapsed/refractory ALL and received preconditioning with cyclophosphamide alone (N = 38, 74%) or in combination with fludarabine (N = 10, 20%), or other (N = 3, 6%). Of the 51 patients who received CD19+ CAR T-cells during the specified period, 34 (68%) developed hyponatremia post infusion (Table 2), and 29 (57%) patients had a decrease in Na > 7 mEq/L post infusion. Of the 34 hyponatremic patients, 13 (38%) had a Na between 133 and 135 mEq/L, 13 (38%) between 130 and 132 mEq/L, and 8 (24%) between 120 and 129 mEq/L. No patients had a Na below 120 mEq/L. 

**Figure 1:**
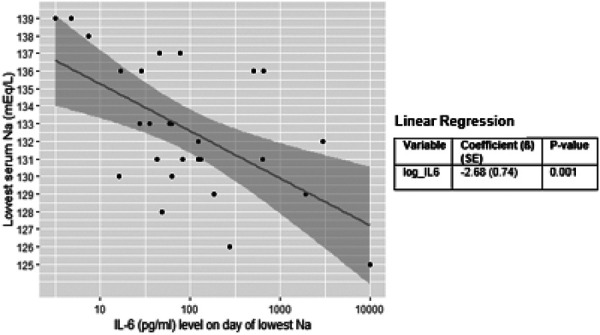
Relationship between IL-6 levels (pg/mL) and lowest serum sodium (mEq/L)).

**Figure 2:**
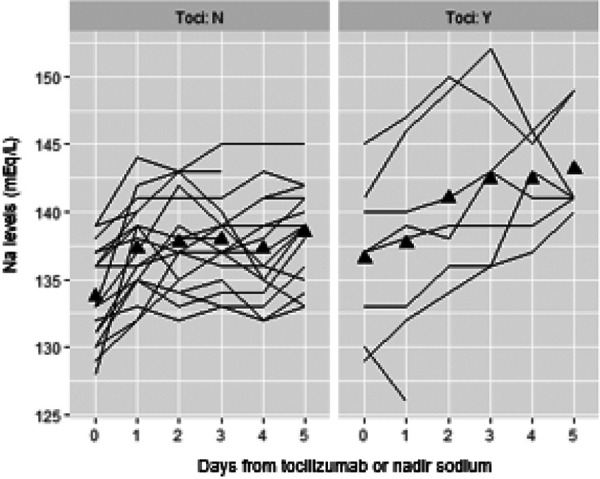
Relationship between tocilizumab administration and serum sodium levels (mEq/L) around the time of the lowest serum sodium.

The median nadir Na value was 133 mEq/L (range 124 – 140 mEq/L). The nadir Na occurred at a median of 5 days (range 1 – 32 days) following CAR T-cell infusion and was not associated with the CAR T-cell dose (p = 0.374). Of the 51 patients, 27 (53%) had an IL-6 level available within 24 hours prior to the nadir Na. Among these 27 patients, there was an inverse linear relationship between IL-6 levels and nadir Na values (p = 0.001) (Figure 1). Per 10-fold increase in IL-6 level, the nadir Na decreased an average of 2.68 mEq/L. A decrease in Na > 7 mEq/L was not significantly associated with higher IL-6 levels. Tocilizumab was administered to 8 of the 27 patients (30%) who had a measured an IL-6 level within 24 hours prior to the nadir Na. The median change in Na within 5 days after tocilizumab administration was 4 mEq/L (range 0 – 12). The change in Na was not significant between those patients who received tocilizumab and those who did not (Figure 2). 

## Discussion 

In this single-center retrospective review, 68% of patients with relapsed/refractory B-cell ALL who received CD19+ CAR T-cells experienced hyponatremia defined as a serum Na < 135 meq/L. Hyponatremia was observed more frequently in this cohort than has been reported in the initial clinical trials for the two currently available FDA-approved CD19+-directed CAR T-cell therapies, axicabtagene ciloleucel (33% for any grade) and tisagenlecleucel (11% for Na < 130 mEq/L) [11]. The rate of hyponatremia in our study is higher compared to these trials. A few potential reasons for this discrepancy could be different co-stimulatory domains, treatment in different disease states (e.g., ALL and non-Hodgkin lymphoma), volume of IV fluids administered, and/or different rates of CRS. Even so, the observed rate of hyponatremia was almost 2-fold greater than in the initial reports. CAR T-cell dose has also been purported to correlate with severity of CRS and IL-6 levels, which was not noted to be statistically significant in this review [8]. 

Hyponatremia is common in cancer patients, with reported rates as high as 47%, depending on the cohort studied and the definition for hyponatremia [12]. Chemotherapy drugs can cause hyponatremia via several mechanisms, including SIADH, dehydration, and much less frequently, salt-wasting nephropathy. Cyclophosphamide (CTX) requires vigorous hydration with intravenous normal saline (IV NS) to prevent bladder toxicity from acrolein and is a common culprit for SIADH-mediated hyponatremia in cancer patients [13]. It is unlikely that the CTX-containing conditioning regimen used in this cohort was responsible for the observed hyponatremia since all patients received CTX at least 2 days prior to CAR T-cell infusion. CTX has a half-life of ~ 12 hours and would have been eliminated after 48 hours [14]. The nadir Na was observed at day 5. 

Studies have shown that IL-6 levels typically rise from the time of CAR T-cell infusion and peak at days 5 – 7 [8]. This timeline is consistent with the significant inverse linear relationship between IL-6 levels within 24 hours of the nadir Na (p = 0.001) and with the nadir Na most frequently being observed at day 5. All patients in this cohort received maintenance intravenous fluids from the time before CAR T-cell infusion. Circulating IL-6 can be transported across the blood brain barrier and stimulate thirst and AVP secretion in the hypothalamus from the supraoptic and paraventricular nuclei [6]. The combination of antidiuresis effects of IL-6-mediated increases in ADH, infusion of IV NS, and increased oral water intake could all have contributed to the high rate of hyponatremia observed in this cohort. 

Tocilizumab is a humanized anti-human IL-6 receptor antibody. Because it binds the IL-6 receptor, IL-6 levels transiently increase following its administration [9]. One would expect that the Na level would decline further after tocilizumab infusion. Only 8 patients in this cohort received tocilizumab, and there was no significant difference in changes in Na values between those who had received tocilizumab and those who did not (Figure 2). This was a small sample size, making it difficult to draw significant conclusions. 

Neurotoxicity due to CRS can range from mild declines in level of alertness to frank seizures and signs and symptoms of increased intracranial pressure. These clinical signs and symptoms can be similar to those observed in the setting of hyponatremia, such as disorientation, inattentiveness, and nausea. While the pathophysiology of CAR T-cell-related encephalopathy remains poorly defined, it seems to have a biphasic course with the first phase occurring around day 5 and the second occurring several days following resolution of CRS [8]. In this cohort, the onset of neurotoxicity was observed at day 5.5 (range 1 – 10 days). This raises the question whether some of the early-phase symptoms of neurotoxicity may reflect symptomatic hyponatremia. 

A thorough evaluation for the etiology of hyponatremia includes analysis of the patient’s volume status, urine electrolytes, thyroid stimulation test, serum cortisol level, and serum and urine osmolarity. The retrospective nature of this study did not allow us to collect and examine all this data. A prospective analysis would provide more insight into the etiology of hyponatremia following CAR T-cell therapy and the relationship, if any, to CAR T-cell-associated neurotoxicity. 

## Conclusion 

Hyponatremia was observed in the majority of patients. The median lowest Na was 133 mEq/L (range 124 – 140 mEq/L) and occurred around day 5 post CAR T-cell infusion. There was an inverse linear relationship between IL-6 and nadir Na (p = 0.001), and per 10-fold increase in IL-6, nadir Na decreased on average 2.68 mEq/L. 

## Funding 

Not applicable. 

## Conflict of interest 

Not applicable. 

**Table 1.** Patient demographics, disease characteristics, and conditioning regimen.

Characteristics	N (%)
Gender	
Male	38 (75)
Female	13 (25)
Age (median years, range)	44 (23 – 74)
Diagnosis	
ALL	51 (100)
Ph (+)	15 (29)
Ph (–)	36 (71)
CD19+ CAR T-cell dose	
4×10^5^	1 (2)
1×10^6^	19 (37)
3×10^6^	31 (61)
Conditioning regimen prior to CD19+ CAR T-cell therapy	
Cyclophosphamide 3,000 mg/m^2^	27(53)
Cyclophosphamide 1,500 mg/m^2^	11 (21)
Cyclophosphamide 3,000 mg/m^2^ + fludarabine 25 mg/m^2^	8 (16)
Cycloposphamide 60 mg/kg + fludarabine 25 mg/m^2^	2 (4)
Cyclophosphamide 3,000 mg/m^2^ + dasatinib 100 mg	2 (4)
Cyclophosphamide 400 mg/m^2^ + clofarabine 20 mg/m^2^	1 (2)

**Table 2.** Incidence of hyponatremia.

Serum Sodium (Na)	N (%)
Hyponatremia (Na ≤ 135 mEq/L)	34 (68)
133 – 135 mEq/L	13 (38)
130 – 132 mEq/L	13 (38)
120 – 129 mEq/L	8 (24)
Change in serum Na > 7 mEq/L	29 (57)
Median lowest serum Na	133 (124 – 140)
